# Array tomography: characterizing FAC-sorted populations of zebrafish immune cells by their 3D ultrastructure

**DOI:** 10.1111/jmi.12223

**Published:** 2015-01-21

**Authors:** Irene Wacker, Peter Chockley, Carolin Bartels, Waldemar Spomer, Andreas Hofmann, Ulrich Gengenbach, Sachin Singh, Marlene Thaler, Clemens Grabher, RASMUS R SCHRÖDER

**Affiliations:** * Centre for Advanced Materials, Universität HeidelbergHeidelberg, Germany; † Heidelberg Karlsruhe Research PartnershipHeidelberg/Karlsruhe, Germany; ‡ Institute of Toxicology and Genetics, Karlsruhe Institute of TechnologyKarlsruhe, Germany; § Institute for Applied Computer Science, Karlsruhe Institute of TechnologyKarlsruhe, Germany; || Carl Zeiss Microscopy GmbHOberkochen, Germany; # Cryo-EM, CellNetworks, BioQuant Universitätsklinikum HeidelbergHeidelberg, Germany

**Keywords:** 3D reconstruction, array tomography, cytotoxic cells, immunological synapse, large volume ultrastructure, zebrafish

## Abstract

For 3D reconstructions of whole immune cells from zebrafish, isolated from adult animals by FAC-sorting we employed array tomography on hundreds of serial sections deposited on silicon wafers. Image stacks were either recorded manually or automatically with the newly released ZEISS Atlas 5 Array Tomography platform on a Zeiss FEGSEM. To characterize different populations of immune cells, organelle inventories were created by segmenting individual cells. In addition, arrays were used for quantification of cell populations with respect to the various cell types they contained. The detection of immunological synapses in cocultures of cell populations from thymus or WKM with cancer cells helped to identify the cytotoxic nature of these cells. Our results demonstrate the practicality and benefit of AT for high-throughput ultrastructural imaging of substantial volumes.

**Lay Description:**

To look at immune cells from zebrafish we employed array tomography, a technique where arrays of serial sections deposited on solid substrates are used for imaging. Cell populations were isolated from the different organs of zebrafish involved in haematopoiesis, the production of blood cells. They were chemically fixed and centrifuged to concentrate them in a pellet that was then dehydrated and embedded in resin. Using a custom-built handling device it was possible to place hundreds of serial sections on silicon wafers as well ordered arrays. To image a whole cell at a resolution that would allow identifying all the organelles (i.e. compartments surrounded by membranes) inside the cell, stacks of usually 50–100 images were recorded in a scanning electron microscope (SEM). This recording was either done manually or automatically using the newly released Atlas Array Tomography platform on a ZEISS SEM. For the imaging of the sections a pixel size of about 5 nm was chosen, which defines membrane boundaries very well and allows segmentation of the membrane topology. After alignment of the images, cellular components were segmented to locate the individual organelles within the 3D reconstruction of the whole cell and also to create an inventory of organelles. Based on their morphologies we could identify specific cell types in the different hematopoietic organs. We could also quantify the proportion of each cell type in the whole population isolated from a given organ.

Some of these specific cells from zebrafish were grown in a culture dish together with human cancer cells. By time-lapse light microscopy we observed that the fish cells attacked the cancer cells and killed them. From this we concluded that these cells must be similar to the cytotoxic cells from humans that play an important role in defence against spontaneously arising cancer cells in our bodies. They form special structures, called immunological synapses that we could also identify on our arrays and reconstruct in 3D. This is the first time the potential of zebrafish immune cells to form immunological synapses has been demonstrated.

Our study is a good example for the practicality and benefit of array tomography in high-throughput ultrastructure imaging of substantial volumes, applicable to many areas of cell and developmental biology.

## Introduction

In view of the recent progress made by using super resolution light microscopy (reviewed in e.g. Schermelleh *et al*., 2010) to address problems in cell and developmental biology, the question arises whether electron microscopy is still delivering additional benefits for these disciplines. Although it is the method of choice to investigate interactions between individual molecules or molecular complexes (Kühlbrandt, 2014; Hoenger, 2014) its impact at the level of cells and tissues seems to be decreasing. In neurobiology, however, a new boost for imaging with electrons arose with the advent of the connectome projects (cp Ultrastructural Brain Mapping Consortium 2014; WIRED DIFFERENTLY 2014). Here the goal is to image whole brains at a resolution that allows identification of synaptic contacts, requiring voxel sizes of better than 5 nm. A number of methods have been developed to achieve that aim comprising FIBSEM (reviewed in Holzer & Cantoni, 2012; Kizilyaprak *et al*., 2014), SBF-SEM (Denk & Horstmann, 2004), but also array tomography (AT, Micheva & Smith, 2007). The latter was initially used predominantly to produce a matrix of serial brain sections arranged on coated glass slides on which antibody labelling could be multiplexed to allow characterization of neurons by fluorescence light microscopy. In the meantime a number of variations on the original theme have extended the method to SEM imaging and also to correlative approaches (mini review Wacker & Schröder, 2013).

Here we show how AT can be expanded to characterize large structures such as whole cells, tissue, or in the future, whole small organisms at the ultrastructural level. As a case study we present a cytotoxic cell-type, which was isolated from adult zebrafish whole kidney marrow (WKM) by FAC-sorting. We used arrays of hundreds of serial sections together with automated high-resolution imaging in an SEM operated with the ZEISS Atlas 5 Array Tomography platform (recently introduced, Carl Zeiss Microscopy GmbH, Germany) to distinguish different cell populations based on their 3D ultrastructure.

Within the last ten years zebrafish has been established as a model system for vertebrate immunity (Trede *et al*., 2004; Carradice & Lieschke, 2008; Meeker & Trede, 2008; Ellett & Lieschke, 2010; Renshaw & Trede, 2012). In adult fish the main hematopoietic organ is the kidney marrow, which is equivalent to the bone marrow in mammals. Here the precursors for the myeloid as well as the lymphoid lineages are produced. Many of the mature cell types found in human blood, such as erythrocytes, thrombocytes, neutrophils, eosinophils and macrophages of the myeloid lineage as well as T cells and B cells of the lymphoid lineage have also been described for zebrafish (Meeker & Trede, 2008). However, unlike the situation in humans, where the different subtypes of blood cells can be identified and sorted by staining with combinations of CD surface markers (Zola *et al*., 2007) such markers are not readily available for zebrafish. Therefore, identification of blood cells isolated from zebrafish is not an easy task. In bright field light microscopy, even after staining, many subtypes, especially in the lymphoid lineage, appear similar (Trede *et al*., 2004; Carradice & Lieschke, 2008). Nonetheless the possibility exists to identify cell types by comparing their ultrastructure with published images (Bennett *et al*., 2001; Lugo-Villarino *et al*., 2010) yet such images are not available for all cell types.

Here we tried to push AT towards high throughput enabling reconstructing of whole cells to learn more about our unknown cell population and therefore facilitate characterization of cell populations isolated from different hematopoietic organs.

## Materials and methods

WKM, spleen and thymus were isolated from five adult zebrafish and placed in L-15 medium (Sigma-Aldrich, Taufkirchen, Germany) with 5% FBS (foetal bovine serum). They were triturated and subsequently passed through a 40-μm filter and centrifuged at 450 *g*. The pellet was resuspended in medium and filtered again (Traver *et al*., 2003). These single cell suspensions were analysed and sorted utilizing a FACS Aria II flow cytometer (BD Biosciences, Heidelberg, Germany). Debris was separated from cells by gating all suspensions on size and granularity based on the forward and side scatter characteristics, respectively.

For coculture experiments cells isolated from WKM or thymus were grown at a 5:1 ratio with a human promyelocytic leukaemia cell line, HL-60, in RPMI medium with 10% FBS and 1% Penicillin and Streptomycin at 37 °C and 5% CO_2_ for 18 hours.

Purified cell populations (25 000–100 000 cells) or cocultures were pelleted and resuspended in 150 μL 0.1 M Pipes, pH 6.8. The resuspended cells were then fixed by adding 150 μL primary fixative (mix of 4% paraformaldehyde, 4% glutaraldehyde in 0.1 M Pipes) and pelleted at 10 000 *g*. The pellet was embedded in 500 μL 2% low melt agarose (Carl Roth, Karlsruhe, Germany) at 60 °C (Bozzola 2014) followed by centrifugation at 14 000 *g*. The resulting stabilized pellet was cut from the agarose block, washed twice in 0.1 M Pipes, postfixed in 0.5% OsO_4_/0.8% K_3_[Fe(CN)_6_] · 3H_2_O (potassium ferricyanide) and block-stained overnight in 2% uranyl-acetate in 25% ethanol. Samples were then dehydrated in a graded ethanol series of 50%, 70%, 90% and 2× 100% ethanol for 10 minutes each, transferred to 100% acetone and infiltrated with 30% and 70% Epon (Serva, Heidelberg, Germany) in acetone for 2–3 hours each. After infiltration overnight in 100% Epon samples were flat embedded in silicone moulds and polymerized at 60 °C for 2 days.

Serial sections were produced on a Leica UC7 ultramicrotome using a Jumbo Knife (Diatome, Biel, Switzerland) and placed on pieces of silicon wafers with the help of a custom built handling device (Spomer *et al*., details to be published elsewhere). They were poststained with 3% uranyl-acetate and Reynolds lead citrate (Reynolds, 1963).

In some cases cells were preselected by imaging the sections with reflected light using a VK-9710K Color 3D laser scanning microscope (Keyence, Neu-Isenburg, Germany).

High-resolution images of serial sections were either acquired manually in an Ultra FEGSEM (Carl Zeiss Microscopy GmbH) at 1.5 kV using the InLens detector or in an automated fashion using the ZEISS Atlas 5 Array Tomography platform (Carl Zeiss Microscopy GmbH) in a Merlin Compact VP FEGSEM at 8 kV using the BSE detector (Carl Zeiss Microscopy GmbH). Using computer-assisted tools in the ZEISS Atlas 5 Array Tomography application serial sections were imaged at multiple resolutions: In the SEM, using light optical images for navigation, serial sections were recorded automatically with 50-nm pixel size. On these section images interesting cells were selected for further high-resolution imaging. These cells were automatically imaged over a range of 100 serial sections with 5-nm pixel size using either a large single frame or a multiimage mosaic for each site.

The resulting image stacks were registered using the stackreg plugin (Thévenaz *et al*., 1998) of Fiji (Schindelin *et al*., 2012). Subsequent volume rendering and segmentation were performed with the Amira software package (VSG/FEI, USA).

## Results

### Establishment of a workflow to create organelle inventories

During studies on innate immunity we isolated a lymphoid cell population marked by expression of GFP under the control of the mpeg1 gene promoter (Grabher, unpublished and Ellett *et al*., 2011) by fluorescence-activated cell sorting (FACS). In adult zebrafish, this cell population did not resemble any of the well-characterized blood cells in zebrafish described so far. To find out what their nature might be, we sought to characterize them by their ultrastructure.

**Figure 1:**
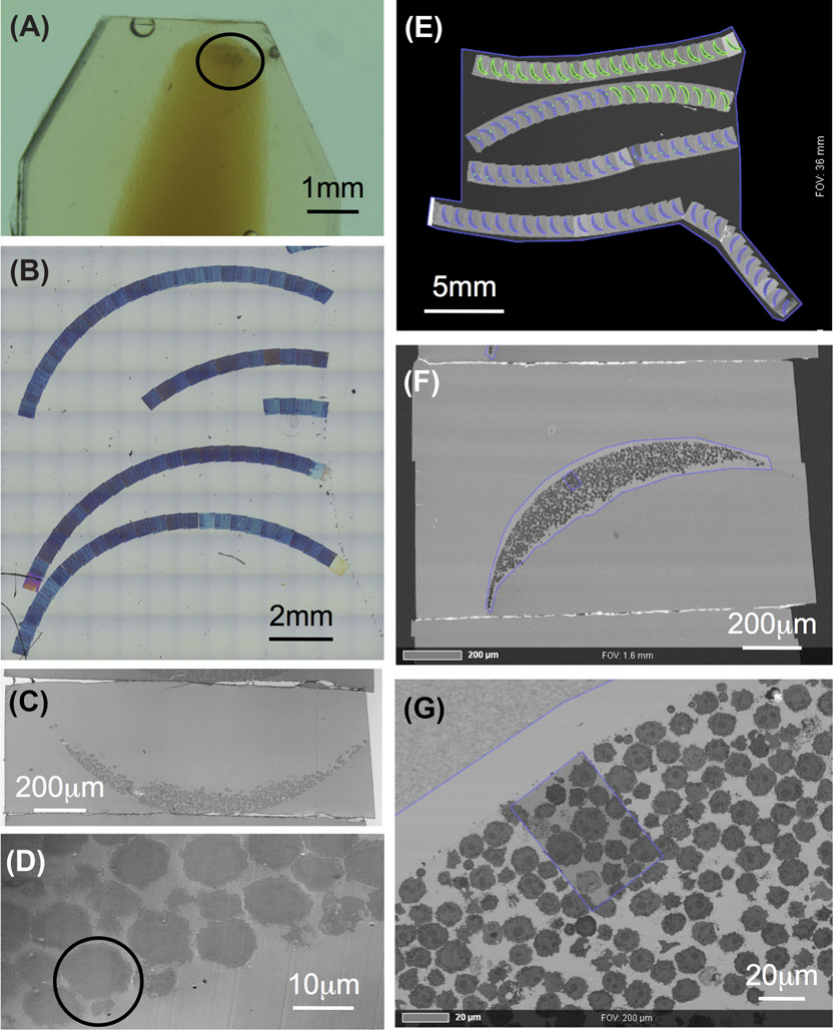
AT workflow: Pellet of isolated zebrafish immune cells (circle) in agarose plug, embedded in epoxy-resin (A), array of 100 nm sections on silicon wafer imaged with reflection light microscopy (B, C, D), single section (C), candidate cell pair (circle) (D). Whole array imaged using Atlas 5 Array Tomography platform (E), single section (F), definition of ROI (G).

In zebrafish the number of purified immune cells representing a given cell-type that can be recovered from one fish organ is on the order of a few thousand cells per organ (Traver *et al*., 2003). Handling such a small number of cells is increasingly difficult. We therefore performed agarose enrobement (Bozzola 2014) to concentrate and stabilize the purified cells during the fixation and embedding procedures. Figure1A shows such an agarose plug inside a polymerized Epon block. The area in the circle contains about 30 000 cells from the spleens of five adult fish.

Collecting images from just a few single sections in the TEM (data not shown) did not allow us to clearly characterize individual cells: a discrete section through a cell will neither contain information about this cell’s extension in 3D nor a representative sampling of the cell’s organelles and its general morphology. To obtain complete information about a cell, one has to do serial section analysis that is extremely time consuming using a TEM. Furthermore, regarding the composition of the different cell populations, inspection of a statistically significant numbers of cells would also be very tedious.

We therefore switched to SEM imaging and to that end produced large arrays of up to 200 serial sections on silicon wafers (Fig.1B). First, utilizing either reflected light microscopy (Figs.1C and D) or the ZEISS Atlas 5 Array Tomography platform (Figs.1E–G), we could preselect specific cells or events, such as the formation of an immunological synapse (circle in Fig.1D).

**Figure 2:**
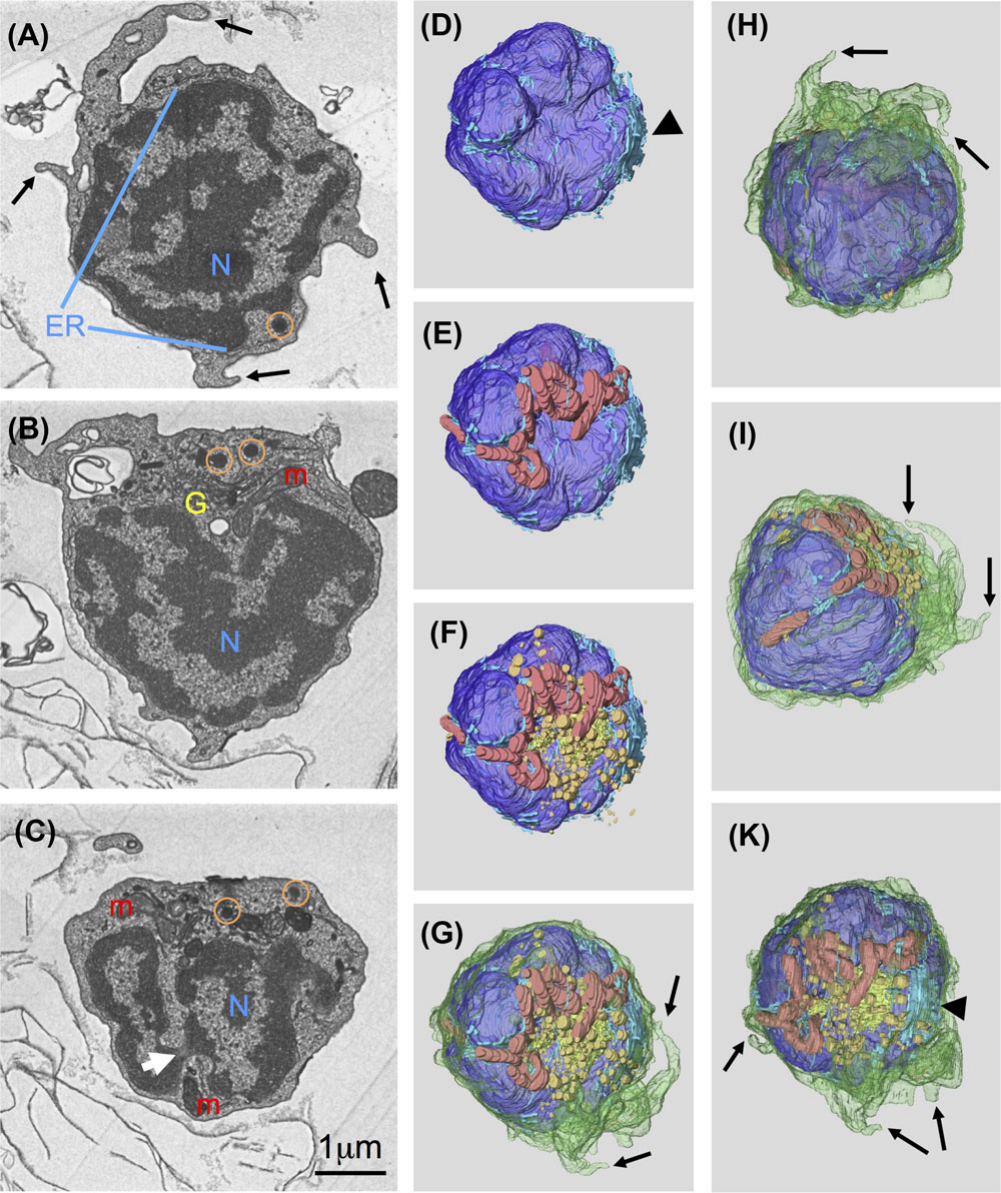
Reconstruction of a whole cell to create inventory of organelles: Sections 17 (A), 37 (B) and 52 (C) from a set of 68 sections (nominal thickness 70nm), white arrow points to narrow constriction of nucleus. 3D representation of volume segmented in Amira (D–K) to illustrate asymmetric distribution of most organelles, colour codes: plasma membrane green, nucleus (N) dark blue, endoplasmic reticulum (ER) light blue, mitochondria (m) red, Golgi complex (G) yellow, lysosomes (circles) orange, black arrowhead points to large ER cisterna, black arrows indicate processes emerging from cell surface.

Certain immune cells contain specific organelles that can easily be recognized. For example, in the case of neutrophils, cigar-shaped electron-dense granules with para-crystalline inclusions (Bennett *et al*., 2001) can be identified. Consequently, we aimed at creating a complete inventory of cellular components by reconstructing whole cells. Figure2 shows representative sections including different aspects of the segmented volume of a typical cell isolated from thymus (for the complete dataset see Movie S1). The presence of a large, deeply lobed (white arrow in Figs.2C and D) nucleus (colour code dark blue) and little cytoplasm is a hallmark of lymphoid cells. There are two or three branched mitochondria (Figs.2B, C, E colour code red) that follow the contours of the nucleus very closely (Figs.2E and I). Several longer and shorter processes and lamellae (black arrows in Figs.2A, G–K) emerge from the cell body. Small cisternae of the ER (colour code light blue) are distributed in varying density throughout the whole volume of the cell’s cytoplasm. One large cisterna (arrowhead in Figs.2D and K) is located to the Golgi complex (colour code yellow). Other vesicular structures are found at one side of the nucleus (Figs.2B, C, F) that often shows a hollow in exactly that region. Secretory lysosomes, identified by their electron dense cores, are also clustered in that region (Figs.2B, C, F, K colour code orange). This strong asymmetry in organelle distribution becomes even more apparent when comparing two opposing views (top view and bottom view) of the subvolume. One is rich in secretory organelles (Fig.2K) whereas the opposite direction (Fig.2H) shows a view where the nucleus appears to fill the whole cell (for a complete 360° viewing see Movie S2).

### Functional imaging – detection of immunological synapses

**Figure 3:**
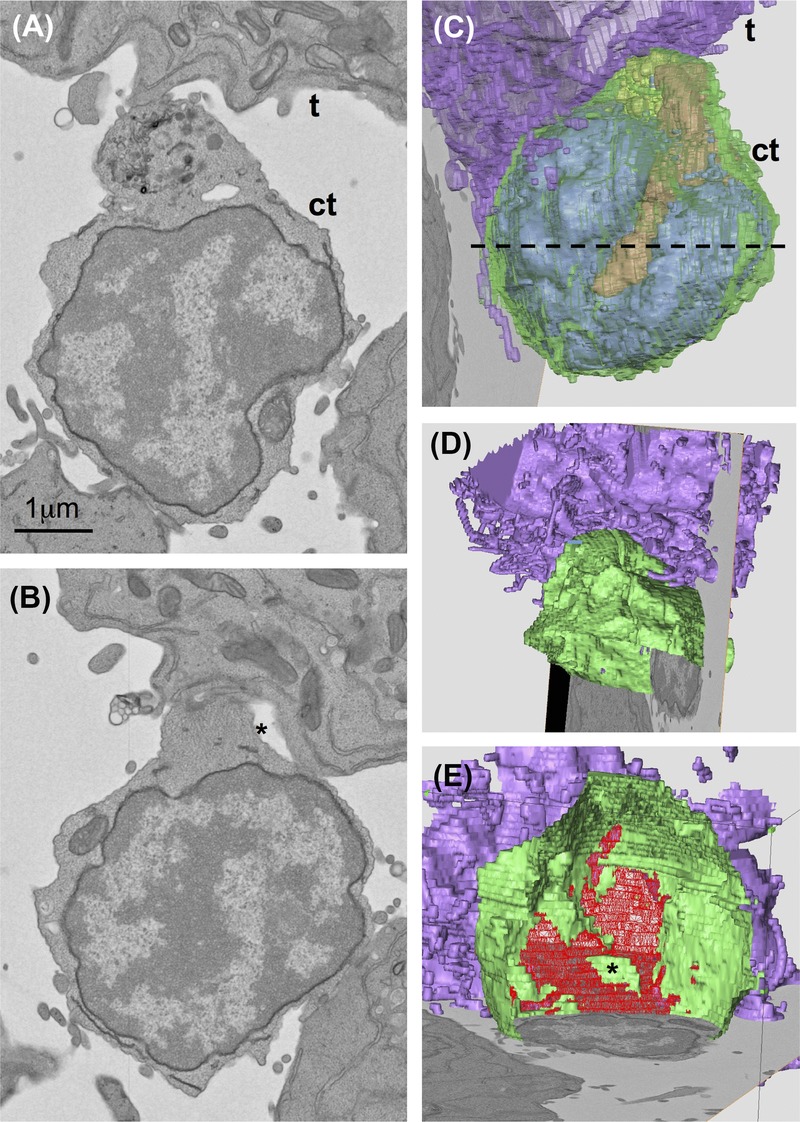
Reconstruction of an immunological synapse: cell population isolated from WKM and cultured with HL60 cell line, cell couple identified on low magnification images of whole sections produced with Atlas 5 Array Tomography platform. Sections 30 (A), 23 (B) from a set of 52 sections (nominal thickness 100nm), t = target cell, ct = cytotoxic cell. 3D representation of volume segmented in Amira (C, D, E) to illustrate relocation of secretory organelles towards target cell (C) and the contact zone (star indicates luminal space) between the two cells (D, E). Dashed line in C shows where cytotoxic cell was cut open virtually for (D) and (E). Colour codes: plasma membrane green, nucleus and endoplasmic reticulum blue, mitochondria red, Golgi complex/ lysosomes yellow/orange, target cell membrane purple, contact zone between both cells patterned in red (E).

In cocultures of zebrafish immune cells originating from thymus or WKM and the HL-60 (human promyelocytic leukemia) cell line we found structures resembling immunological synapses (Fig.3). Our findings are comparable to earlier results described by TEM for the interaction of cytotoxic T lymphocytes (Stinchcombe *et al*., 2006) or natural killer cells (Stinchcombe *et al*., 2011) with their targets. We could demonstrate by collecting time-lapse imaging light microscopy data that these cell populations can indeed act as cytotoxic cells and kill their targets (manuscript in preparation). Figures3A and C show the typical accumulation of Golgi apparatus, lytic granules and other vesicular structures close to the contact site between the target and the cytotoxic cell. The formation of tight contacts between the plasma membranes of both cells enclosing a luminar space (star in Figs.3B and E) between them is illustrated in Figure3B. Here and also in Figures3C and D a nozzle-like protrusion of the cytotoxic cell towards the target becomes evident (see also Movie S3 for the whole image stack including segmentation). The cytotoxic cell is cut open virtually along the dashed line in Figure3C to better visualize the membrane contacts. Figure3E shows an alternative view rotated by 180°. Looking into the opened cytotoxic cell (Fig.3E, Movie S4) reveals the contact area to the target cell patterned in red. The green part inside the red represents the lumen between the two cells seen in Figure3B (star) in cross-section.

### Using arrays of serial sections for quantification and statistics

**Figure 4:**
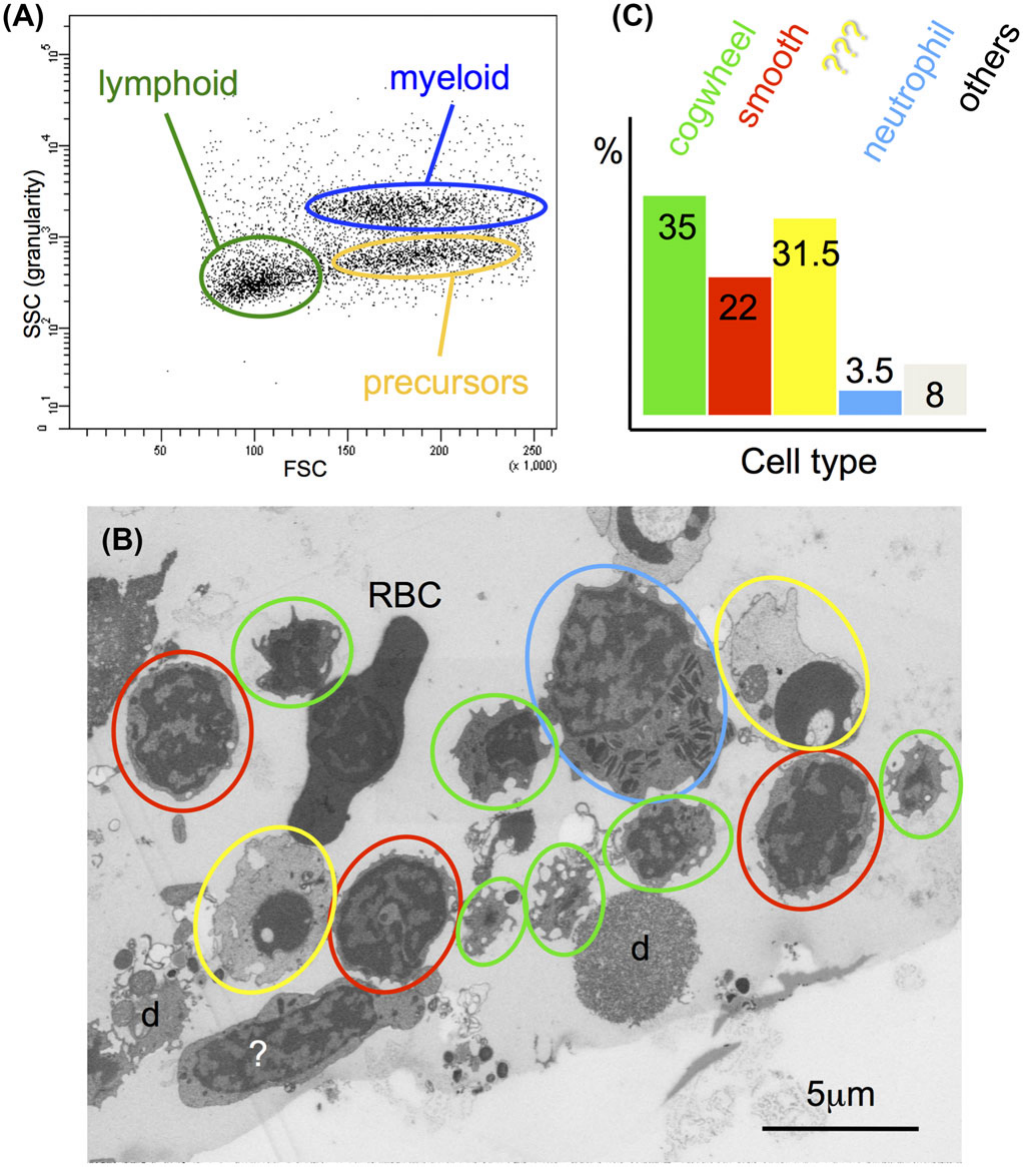
Quantification of the different cell types found in cell populations isolated from WKM: FACS plot (A) illustrating the three main populations found in that organ when sorting according to forward scatter (FSC) and side scatter (SSC). Overview (B) illustrating different morphologies found in the lymphoid gate: small cells with ruffled surface (green circles), small cells with smooth surface (red circles), large cells containing cigar-shaped granules (blue circles), cells with condensed nucleus (yellow), red blood cells (RBC) and debris (d) plus some other unidentified cells (?). (C) Percentage of different classes identified in (B) based on 240 cells counted in three different sections, ca. 5 μm apart from each other in *z*-direction.

In addition to creating organelle inventories and the first 3D reconstruction at ultrastructural resolution of a complete immunological synapse between a cytotoxic cell and its target we used the arrays of sections also for quantification of cell distributions. FAC-sorted cell populations are not 100% pure. Looking at such a diverse population indeed reveals a number of different morphologies. An example of cells isolated from the lymphoid gate of WKM is given in Figure4A. Even with a tiny pellet (cp, Fig.1A) containing only a relatively small number of ‘good’ cells in one section, arrays containing hundreds of serial sections allow looking at a sufficiently high number of cells for statistical analysis: whole sections are recorded at a resolution adequate for recognizing different cell types. The next section to be recorded is chosen according to cell size in such a way that prevents the same cell from being imaged twice. From the population shown in Figure4B, 240 cells were assessed in terms of size and morphology (Fig.4C). Neutrophils (colour code blue) are easily recognized by their elongated, electron dense granules and are very scarce in the lymphoid population. The nature of the second largest population (colour code yellow), exhibiting a very condensed nucleus within very light, unstructured cytoplasm, is unclear. They look similar to the population of very small embryonic-like stem cells isolated from adult murine bone marrow (Kucia *et al*., 2006) whose true identity is still under discussion (Abbott, 2013).

**Figure 5:**
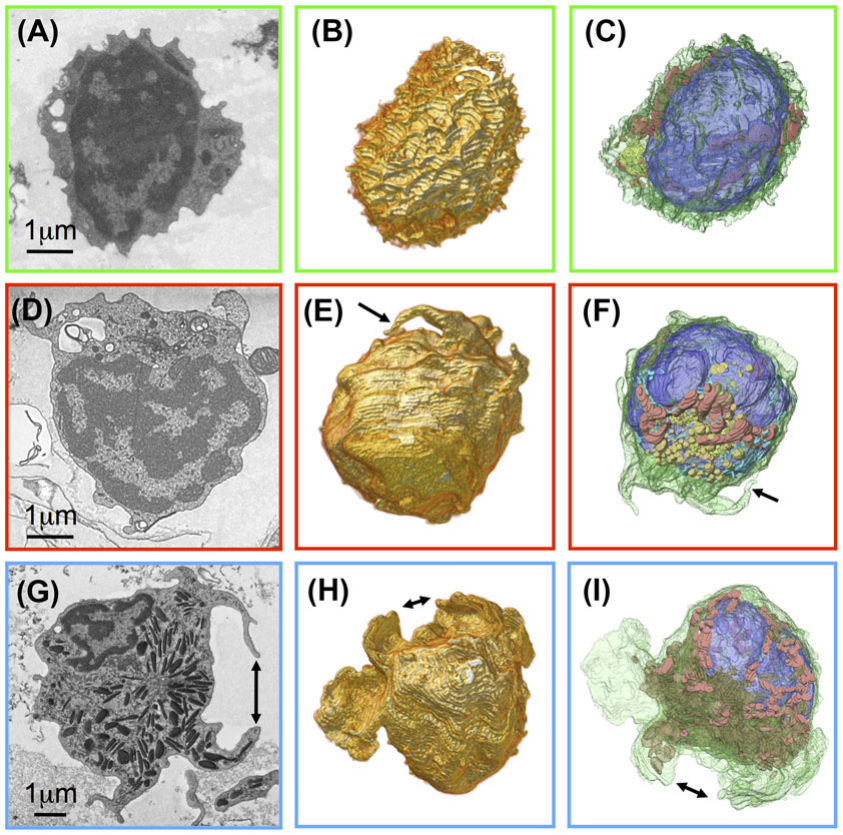
Major cell types found in population isolated from adrenal medulla: Two different predominant morphologies, cogwheels (A–C) and smoother with longer processes (D–F), without azurophilic granules – compare with neutrophil which also has long processes/lamellae and lots of granules (G–I). Single sections (A, D, G), volume rendering of cell surfaces (B, E, H), segmentation of plasma membrane (green), nucleus (blue), mitochondria (red), Golgi complex (yellow), secretory lysosomes (orange), azurophilic granules (brown). Double arrows mark corresponding lamellae, single arrows corresponding process in the different representations.

The other two large populations, looking like smaller cogwheels or larger Easter eggs respectively, are highlighted by colour codes green and red. They were present at different proportions in all three hematopoietic organs we investigated (WKM, thymus, spleen) and are tentatively classified as cytotoxic cells by us. Since the cross sections of these two cell types differ distinctly in their surface morphology we also investigated their surfaces utilizing volume renderings (Fig.5). The cogwheel-shaped cells are a bit smaller and their surface is rippled (Fig.5B) whereas the ones with a smoother surface are somewhat larger and tend to have a few, but extended processes (Figs.5D and E). The inventory of organelles is rather similar for both cell types: a large, lobed nucleus surrounded by a narrow sheath of cytoplasm containing a few mitochondria, ER and one region where Golgi apparatus and secretory lysosomes are clustering (Figs.5C and F). The third cell type shown here (Figs.5 G–I) seems to have long processes also when looking at single sections, however, in 3D it becomes obvious that these are long protruding lamellae (Fig.5H) in reality. In addition, these cells contain a huge number of elongated, electron-dense granules, often incorporating paracrystalline inclusions, typical for fish neutrophils (Figs.5G and I). The identity of this cell type was confirmed by embedding a population of cells isolated from the myeloid gate containing almost 100% neutrophils (data not shown).

## Discussion

One big advantage of AT is the potential to reconstruct volumes considerably larger than those addressable by, for example, FIB-milling (Holzer & Cantoni 2012). This is one reason why many connectome projects (Ultrastructural Brain Mapping Consortium, 2014; WIRED DIFFERENTLY, 2014) rely on AT in one form or another. In that respect we consider here arrays produced by the ATUMtome (Hayworth *et al*., 2014) as a variation of the original AT. Common to all these projects is a very clear question, that is, to find out how the billions of neurons in a brain are connected. This of course can only be answered by imaging whole brains. In cell or developmental biology, however, the questions to be addressed are much more diverse. In many cases it is not necessary to image a large volume in its entirety. A much smaller subvolume may contain the only really interesting part. This could be a specialized structure within a tissue such as a neuromuscular junction (Wacker *et al*., 2010) or a rare event such as the formation of an immunological synapse as we have shown here. Producing arrays of hundreds of sections and imaging them in an automated fashion using an SEM allows screening large numbers of cells for the event of interest. The ultrastructural details of the immunological synapses we observed for the interaction of a newly identified cytotoxic cell from zebrafish resemble those described by electron tomography in a TEM for human or murine cytotoxic T lymphocytes (Stinchcombe *et al*., 2006) or human natural killer cells (Stinchcombe *et al*., 2011).

In a previous report, nonspecific cytotoxic cells (NCC) from zebrafish have been isolated from coelomic cavity exudates after intracoelomic injection of bacteria. They were described (Moss *et al*., 2009) as small lymphocyte-like cells with a high nucleus:cytoplasm ratio, smooth ruffled plasma membrane and small cytoplasmic granules. These findings agree roughly with the one cell type we identified in sorts from WKM, thymus and spleen. However, Moss *et al*. (2009) demonstrated the cytotoxic capacity of their exudate cell population by a redirected lysis assay and not by their ultrastructural morphology like we did. Thus, our 3D reconstruction of a whole cytotoxic cell from zebrafish interacting with its target is the first visualization at the ultrastructural level of this cell type in action.

Another advantage of AT is its potential for statistics: Analysis in 3D reveals differences and characteristics ultimately providing statistical data not obtainable from small volume imaging. As an example we characterize different cell types from an unknown population isolated from WKM by investigating their 3D ultrastructural morphology. Accordingly, once a few cells of a certain phenotype have been reconstructed in 3D, full reconstructions are no longer necessary to identify a given cell type. In special cases even a single 2D profile may be sufficient for classification, thus allowing the quantification of large numbers of cells for statistical analysis.

The possibility to use a given array repeatedly and for different purposes is another definite advantage of AT over destructive methods for 3D reconstructions using an SEM such as FIBSEM or SBF-SEM/3View (Denk & Horstmann, 2004; Gatan Inc. Pleasanton, CA, USA), where imaging is done on the blockface which has to be freshly exposed for each image resulting eventually in the irretrievable loss of the sample material. In view of the sparseness of material that can be obtained from the hematopoietic organs of a single adult zebrafish (e.g. one spleen contains fewer than 100 000 leucocytes total, Moss *et al*., 2009), AT is clearly the method of choice that facilitates optimal usage of precious material.

One disadvantage of AT compared to FIBSEM is its sampling property which is discrete and thus leads to anisotropic voxels. This can easily be seen in our reconstructed volumes: they show steps due to the specific acquisition procedures, that is, in the particular SEM imaging modes (unlike in TEM tomography) only the very top layer of a section is imaged. The remaining volume of the section is not used for image formation. Therefore, the steps depend on the section thickness and represent the distance between the consecutive imaging layers. On the other hand, for our purposes in this study a finer sampling was not necessary and would have increased the amount of data considerably. In conclusion, merging semiautomated handling of sections with automated recording of images enabled us to reconstruct a sizeable number of 3D volumes of individual cells. These new methods exposed another notable bottleneck: the segmentation and annotation of large amounts of data is still a largely manual process. Improved and especially automated tools for analysis and segmentation are needed for the future to uncover all the information we are now able to produce with AT and similar methods.

## Supporting Information

Disclaimer: Supplementary materials have been peer-reviewed but not copyedited.
